# KRAS Gene Copy Number as a Negative Predictive Biomarker for the Treatment of Metastatic Rectal Cancer With Cetuximab: A Case Report

**DOI:** 10.3389/fonc.2022.872630

**Published:** 2022-05-26

**Authors:** Qunli Xiong, Zhu Zeng, Yang Yang, Ya Wang, Yongfeng Xu, Ying Zhou, Jinlu Liu, Zhiwei Zhang, Meng Qiu, Qing Zhu

**Affiliations:** ^1^ Department of Abdominal Oncology, West China Hospital, Sichuan University, Chengdu, China; ^2^ Department of Radiology, West China Hospital, Sichuan University, Chengdu, China

**Keywords:** Kirsten Rat Sarcoma Viral Oncogene Homolog (KRAS), gene copy number, biomarker, colorectal cancer, cetuximab, anti-EGFR monoclonal antibody

## Abstract

**Background:**

Close to one third of colorectal cancer (CRC) patients are diagnosed with metastatic CRC (mCRC). Patients with wild-type RAS and BRAF usually receive anti-EGFR monoclonal antibody therapy containing cetuximab. Overall, 30–50% of mCRC patients are reported to harbor RAS mutations, and RAS mutation status should be assessed when considering EGFR inhibitor treatment according to mCRC biomarker guidelines. Of note, 0.67–2% of patients with CRC harbored a KRAS amplification. Here we reported a case of advanced rectal cancer with wild-type RAS and BRAF in a male patient who harbored a KRAS amplification during anti-EGFR treatment.

**Case Presentation:**

A 46-year-old man was diagnosed with rectal adenocarcinoma with liver metastases (cT3NxM1a, stage IVA). After receiving first-line irinotecan- fluorouracil chemotherapy (FOLFIRI) plus cetuximab, second-line capecitabine- oxaliplatin chemotherapy (XELOX) plus bevacizumab, and third-line regorafenib, he rechallenged FOLFIRI and cetuximab for seven cycles, achieving a prolonged survival of at least 5 months. The KRAS copy number of circulating tumor DNA (ctDNA) was assessed during treatment. Notably, apart from serum carbohydrate antigen 199 (CA199) and carcinoembryonic antigen (CEA), the change of plasm Kirsten Rat Sarcoma Viral Oncogene Homolog (KRAS) copy number appeared to strongly correlate with treatment response.

**Conclusion:**

Our findings suggest that the dynamic change of KRAS copy number on ctDNA during treatment might be a negative predictive biomarker. Additionally, RAS and BRAF wild-type mCRC patients who are resistant to first-line FOLFIRI plus cetuximab therapy may respond well to the FOLFIRI plus cetuximab “rechallenged” strategy.

## Introduction

Colorectal* *cancer (CRC) is one of the most common malignancies, with more than 1.9 million new CRC cases and 935,000 deaths in 2020 (1). A total of 22% of patients with CRC have progressed to metastatic CRC (mCRC) at the time of diagnosis. And the prognosis of mCRC is dismal, with a 5-year survival rate of less than 20% (2). Systemic therapy (cytotoxic chemotherapy, biologic therapy, immunotherapy) is the primary treatment for unresectable mCRC (3, 4). Specifically, the combination of an anti-epidermal growth factor receptor (anti-EGFR) drug with chemotherapy is recommended as the first-line therapy for patients with RAS and BRAF wild-type mCRC (5, 6). After an initial response, secondary resistance to EGFR antibodies limits its application. Genes, including RAS, BRAF, and PI3K downstream of the EGFR signaling pathway, are key regulators of cell proliferation, differentiation, and division (7). And mutations of those genes may result in abnormal activation of the EGFR signaling pathway, contributing to anti-EGFR monoclonal antibody resistance (8–10). Furthermore, the curative effect of EGFR antibodies correlates with tumor sidedness (11, 12). In detail, the poor prognosis of right-sided CRC correlates with frequency alterations of the RAS, BRAF, PI3K, and TGF-β pathways. As for left-sided tumors, amplifications of receptor tyrosine kinases and mutations of APC and TP53 genes are more frequent; in addition, high sensitivity to EGFR antibodies is related to high expression of EGFR ligands amphiregulin (AREG) and epiregulin (EREG) (13–15). However, little is known regarding patients with CRC harboring KRAS copy number variation, and data on the response to anti-EGFR therapy are scarce. Here, we presented a case of an advanced CRC patient who harbored an elevated RAS copy number of ctDNA at the time of progressive disease, and experienced a favorable response to the rechallenge of irinotecan- and cetuximab-containing therapy after failure of multi-line treatment.

## Case Presentation

**Figure 1:**
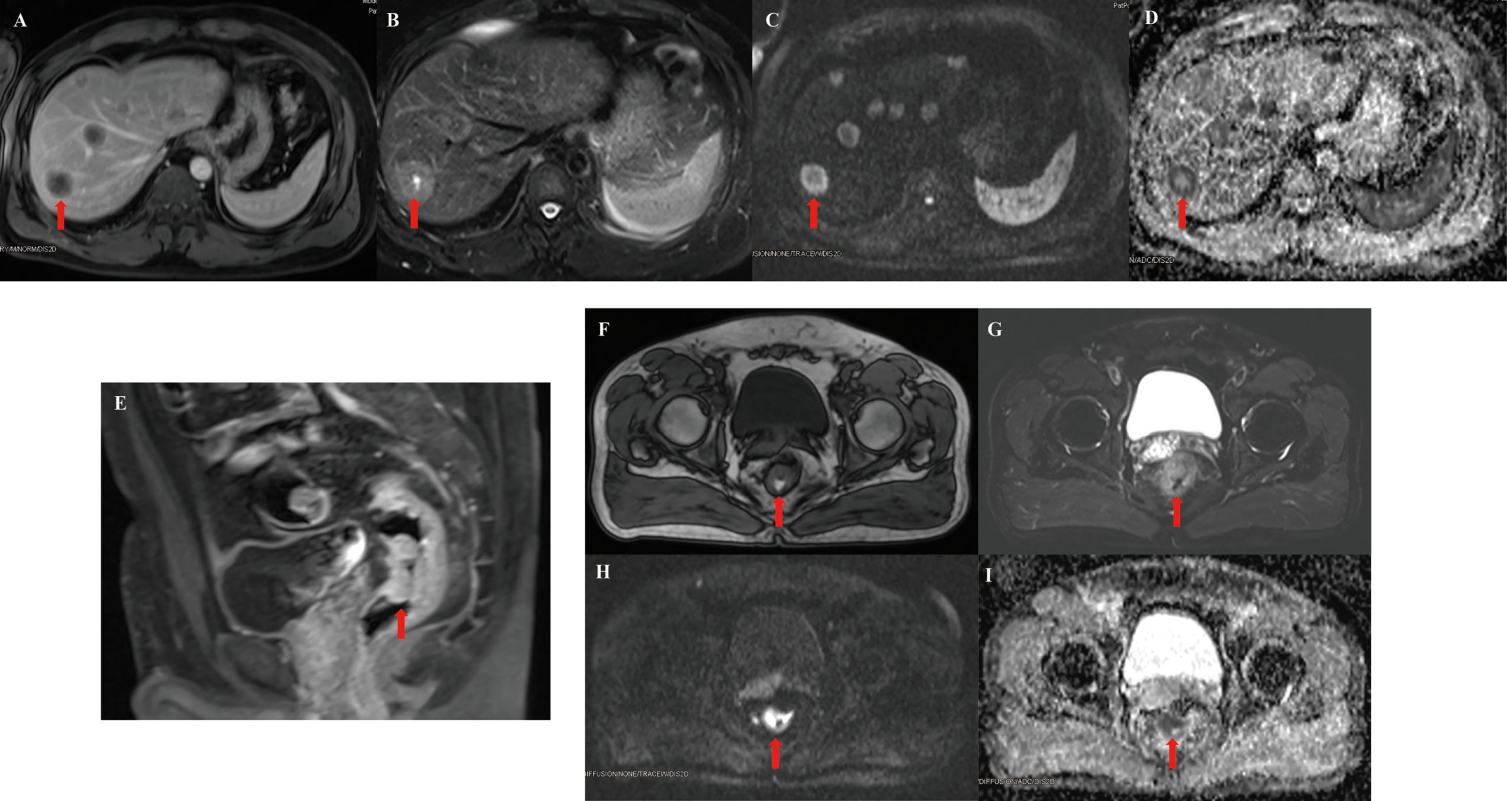
Representative magnetic resonance imaging (MRI) images of lesions. **(A–D)** Representative images of T1-weighted imaging (T1WI), T2-weighted imaging (T2WI), diffusion-weighted imaging (DWI), and apparent diffusion coefficient (ADC) in the liver. **(E–I)** Representative images of rectum lesions in the sagittal position and coronal position (T1WI, T2WI, DWI and ADC), respectively. Red arrows indicate typical liver or rectum lesions.

In February 2018, a 46-year-old man was admitted to our center with chief complaints of increased stool frequency and occasional bloody stool lasting for over 1 month. A neoplasm with a distance of 4 to 7 cm from the anus was found in his colonoscopy, and liver multiple occupancies were detected in his abdominal computed tomography (CT). Subsequently, the patient was diagnosed with middle rectum adenocarcinoma with liver metastasis after conducting a pathological biopsy and magnetic resonance imaging (MRI) of the liver and rectum (**Figure 1**), accompanied with elevated CA199 (>1000.00 U/ml) and CEA (229.70 ng/ml), suggesting a stage of cT3NxM1a, stage IVA according to National Comprehensive Cancer Network (NCCN) staging criteria. He had been generally fit, except for 5 years of chronic gastritis.

**Figure 2:**
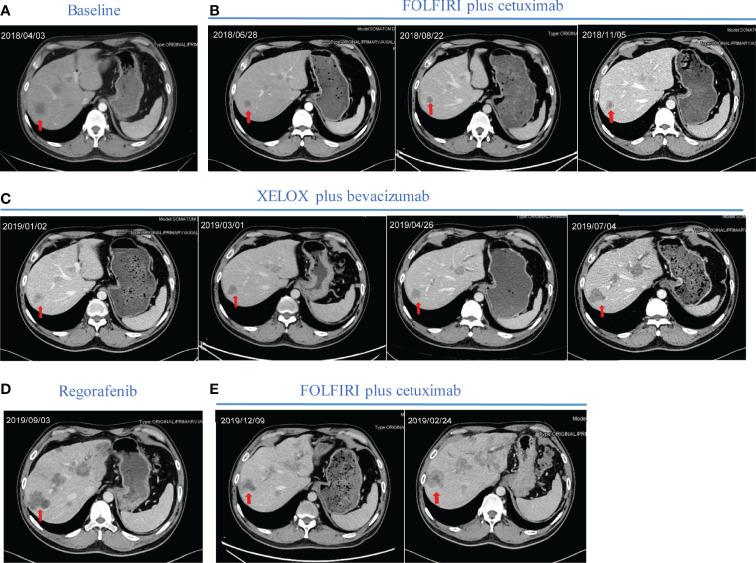
Representative computed tomography (CT) images of liver lesions during treatment. **(A)** Before first-line treatment, the representative CT image of the liver. **(B)** Regular CT was used to assess treatment efficacy during first-line treatment containing FOLFIRI plus cetuximab. **(C)** Regular revision CT was used to assess treatment efficacy during second-line treatment containing XELOX plus bevacizumab. **(D)** CT image of liver lesions after two cycles of regorafenib. **(E)** CT images of liver lesions after two cycles and four cycles of rechallenging FOLFIRI plus cetuximab. Red arrows indicate typical liver lesions. CT computed tomography; FOLFIRI, chemotherapy regimen containing irinotecan and fluorouracil; XELOX, chemotherapy regimen containing capecitabine and oxaliplatin.

**Figure 3:**
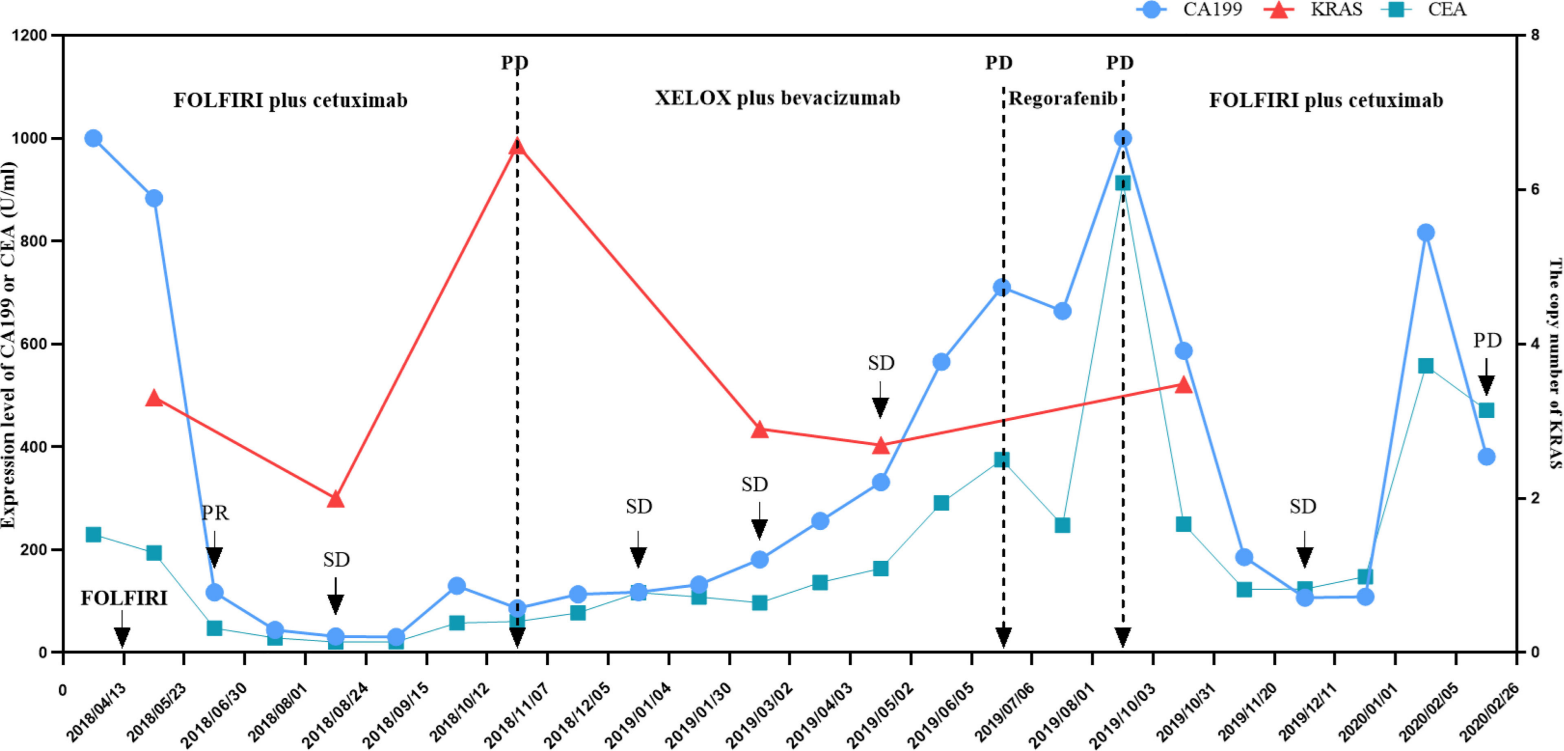
Changes in KRAS copy number, CA199, and CEA level during anti-tumor treatment and follow-up of the patient. The KRAS copy number was measured in the patient’s circulating tumor DNA. The CA199 and CEA tumor markers were measured in the patient’s serum at periodic intervals throughout the clinical course and annotated with the date, therapeutic approach, and treatment efficacy. CA199, cancer antigen 199; CEA, carcinoembryonic antigen; SD, stable response; PR, partial response; FOLFIRI, chemotherapy regimen containing irinotecan and fluorouracil; XELOX, chemotherapy regimen containing capecitabine and oxaliplatin.

Based on the standard principles, the patient initially received first-line chemotherapy with FOLFIRI. No obvious adverse events were observed during chemotherapy. Then target region sequencing concluding 1406 targeted genes for his plasm ctDNA was conducted by NovaSeq, indicating that RAS and BRAF mutations were absent while the KRAS copy number of 3.31 copies was abnormal. Additionally, the status of microsatellite instability (MSI) or mismatch repair (MMR) was defined as proficient mismatch repair (pMMR). Therefore, one anti-EGFR monoclonal antibody (mAbs), cetuximab, was added. After two cycles of therapy, his tumor shrank remarkably, and partial response (PR) was assessed (**Figures 2A, B**), followed by stable disease (SD) based on Response Evaluation Criteria in Solid Tumors (RECIST); simultaneously accompanying with reduced CA199 (30.73 U/ml) and CEA values (21.18 ng/ml) in blood after four cycles. However, disease progressed in November 2018, mainly referring to the liver metastasis (**Figure 2B**). Of note, before CT scanning, the level of CA199 decreased while the copy number of wild-type KRAS and APC p.R499* (a gene encoding tumor suppressor adenomatous polyposis coli (APC) protein) mutation increased (**Supplementary Table 1**), indicating the potential resistance to cetuximab in combination with chemotherapy. From November 2018 to June 2019, the patient then received second-line XELOX chemotherapy plus bevacizumab. During six cycles, his metastasis lesion size in the liver remained stable, and even shrunk (**Figure 2C**), with the copy number of KRAS decreasing to 2.69 copies and mutant frequency of APC p.R499* decreasing to 8.9% (**Supplementary Table 1**). After finishing the second-line therapy, abdominal enhanced CT scanning showed that the size of the liver lesion had enlarged (**Figure 2C**). The patient was subsequently treated with third-line regorafenib. Unfortunately, it failed to control the tumor growth (**Figure 2D**). Then, we decided to choose FOLFIRI chemotherapy combined with cetuximab again after a discussion. On 4 October 2019, his last genetic testing reports showed the copy number of KRAS was 3.48 copies, and the mutant frequency of APC p.R499* was 15.1% (**Supplementary Table 1**). And tumor markers of CA199 and CEA decreased gradually. Although his liver lesions enlarged and the number of lesions also increased after six cycles, the physician considered that it might be due to the prolonged treatment interval (**Figure 2E**). Thus, he received one cycle of FOLFIRI plus cetuximab again. The patient decided to stop the treatment and never returned to the hospital after the last outpatient follow-up in March 2020. **Figure 3** presents the whole process and dynamics in tumor-related markers.

## Discussion

Anti-EGFR mAbs involving cetuximab and panitumumab are critical and routine drugs for patients with RAS and BRAF wild-type mCRC, achieving a median overall survival (OS) of approximately 30 months (16–18). Following the initial response, secondary resistance invariably occurs, thereby limiting the clinical benefit of EGFR antibodies (10). To date, the mechanisms of acquired resistance have been extensively studied. In detail, several studies described that EGFR mutation (19, 20) or downregulation of signal pathways including KRAS-MAPK (21), Janus kinase (JAK)/signal transducer, and activator of transcription (STAT), might be the underlying mechanism of cetuximab resistance (22). Also, aberrant regulation of miRNAs (23) and several proteins (24, 25) has been found to be responsible for cetuximab-induced resistance in CRC. Resistance to first-line cetuximab plus chemotherapy in our case might be due to an elevated copy number of wild-type KRAS, which was consistent with previous studies (26–28).

KRAS and BRAF mutations in colorectal cancer have been widely considered to be associated with patient prognosis (29). Notably, copy number changes at critical regions have shown potential practical utilities predicting treatment response and clinical outcomes (30). Amplification of the KRAS gene has been identified as a crucial factor that leads to RAS/mitogen-activated protein kinase activation (31). Intriguingly, loss of KRAS gene copy number in tumor DNA is associated with better treatment response to anti-EGFR drugs even in the presence of KRAS mutation in the tumor, while amplification of KRAS predicts resistance to drugs independent of mutational status (32). In this paper, the KRAS copy number was elevated when PD was assessed during the first-line treatment of the patient, and vice versa, indicating that the dynamic trends of the KRAS copy number may be a negative predictive biomarker.

Furthermore, we found that mutations in the APC gene, a tumor suppressor that has a functional role in the typical (β-catenin-dependent) Wnt signaling pathway (33), was the most frequent in this case. APC mutations normally promote cell migration by reducing cell adhesion *via* deregulation of β-catenin and E-cadherin distributions among the cytoplasm and the cell membrane (34, 35). Previous research revealed that a portion of 80% of CRC tumors harbor somatic inactivating mutations in the APC gene during the early stages of non-hypermutated CRC (36, 37).

Recently, the potential efficacy of rechallenge with anti-EGFR mAbs in a later setting for patients who were previously treated with EGFR blockades has been suggested in retrospective and prospective studies (38–49) (**Table 1**). The CRICKET trial, a single-arm phase II trial of rechallenge with cetuximab in 28 patients with a response to previous EGFR inhibitions showed an objective response rate (ORR) of 21% and a median PFS of 3.4 months (44), while another phase II trials from Japan (JACCRO-CC-08 and -09) demonstrated limited efficacy of that similar regimen, with an ORR of 2.9-8.3% and median PFS of 2.4-3.1 months (50). Furthermore, D. Santini et al. reported a higher ORR (53.8%) and PFS (6.6 months) (39). And other clinical studies, such as REMARRY and PURSUIT, are ongoing (51). Altogether, rechallenge with anti-EGFR mAbs is feasible in patients with RAS and BRAF wild-type mCRC. In our study, the patient tolerated therapy well and obtained a favorable PFS with 5 months, without any severe adverse events (only red rashes appeared on his face and back). It should be noted that the PFS in our paper was superior to that in current trials (almost 2-4 months) as shown in **Table 1**.

**Table 1:** Literature of reported clinical trials of rechallenge with anti-epidermal growth factor receptor (EGFR) therapy with panitumumab or cetuximab-based therapy for patients with metastatic colorectal cancer.

First author	Published year	Nation	Type of research	Number of patients	Median OS (months)	Median PFS (months)	ORR	DCR
Muhammad Wasif Saif (25)	2010	USA	Retrospective	15	–	4	–	40.0%
D. Santini (7)	2012	Italy	Prospective (phase II)	39	–	6.6	53.8%	89.7%
Raymond C. Wadlow (26)	2012	USA	Prospective (phase II)	20	1.7	5.2	0.0%	45.0%
Filippo Pietrantonio (27)	2013	Italy	Retrospective	30	9.6	4.2	30.0%	67.0%
X. Liu (28)	2015	USA	Retrospective	89	–	4.9	–	58.0%
HIROAKI TANIOKA (33)	2018	Japan	Retrospective	14	–	4.4	21.4%	71.4%
H. Osawa (29)	2018	Japan	Prospective (phase II)	33	8.7	2.9	15.6%	56.2%
Chiara Cremolini (8)	2019	Italy	Prospective (phase II) CRICKET	28	9.8	3.4	21.0%	54.0%
Daniele Rossini (30)	2020	Italy	Retrospective	86	10.2	3.8	19.8%	–
Li Chia Chong (32)	2020	Australia	Retrospective	22	7.7	4.1	4.5%	45.4%
Yu Sunakawa (31)	2020	Japan	Prospective (phase II) JACCRO CC-08 and JACCRO CC-09	58	–	2.4-3.1	2.9%-8.3%	–
Amanda Karani (34)	2020	Brazil	Retrospective	17	7.5	3.3	18.0%	–

OS, overall survival; PFS, progression-free survival; CI, confidence interval; ORR, objective response rate; DCR, disease control rate; -, not mentioned in the literature.

Although retreatment with EGFR inhibitors seems to be beneficial, it is essential to select an appropriate biomarker to monitor disease progression and evaluate response to therapy, such as liquid biopsy. Given its feature of minimal invasiveness and convenience, liquid biopsy has recently attracted interest in the molecular cancer-diagnosis area. For example, the detection of ctDNA in peripheral blood has shown great potential in the clinic, particularly for those patients who cannot undergo biopsy (52–54). Recent research found that in patients with advanced non-small cell lung cancer, elevation of ctDNA preceded an abnormal radiographic finding and the frequency of mutated alleles in ctDNA increased consecutively for 3-5 months before clinical evidence of disease progression (55). Moreover, as for melanoma patients, an undetectable ctDNA at baseline or during treatment tended to correlate with a better objective response to therapy (56). Of note, scholars have identified the KRAS mutation as a driving factor of cetuximab-induced acquired resistance in CRC, which suggested that detecting KRAS mutant clones *via* a non-invasive method (generally peripheral blood sampling) could reflect true tumor performance before radiographic progression (26). However, we did not discover the relation between the KRAS mutant and radiographic progression due to limited and irregular gene testing. Interestingly, the KRAS copy number was elevated at the time of radiographic progression while the level of CA199 and CEA was still low (**Figure 3**), indicating that the status of RAS may be helpful to monitoring therapeutic efficacy. Nevertheless, more evidence is needed to support this phenomenon.

## Conclusion

Our results demonstrated that the elevated KRAS copy number of ctDNA was correlated with progressive disease, and resistance to anti-EGFR treatment might be due to an increased KRAS gene copy number. If economic conditions permit, apart from RAS mutational testing, RAS copy number testing should be performed when considering EGFR inhibitor treatment, especially when the most commonly used clinical tumor markers including CA199 and CEA are at a low level. In addition, we reported an mCRC patient with wild-type KRAS, previously treated with an anti-epidermal growth factor receptor-based regimen, who obtained a good response after rechallenge with cetuximab-based therapy, and achieved a PFS of 5 months. This impressive response highlights the potential efficacy of reintroducing cetuximab for patients with acquired resistance to a previous treatment with chemotherapy plus cetuximab. We expect that our report can provide a reference for the systematic treatment, monitoring, and prognosis determination of patients with RAS and BRAF wild-type mCRC.

## Data Availability Statement

The datasets presented in this study can be found in online repositories. The names of the repository/repositories and accession number(s) can be found below: https://www.ncbi.nlm.nih.gov/, PRJNA790712.

## Ethics Statement

Written informed consent was obtained from the individual for the publication of this case report and any potentially identifiable images or data included in this article.

## Author Contributions

QX and ZZ conceived the study, performed the literature research, wrote the paper, and assessed figure and tables. YY, YW, and YX performed the literature research and critically reviewed the paper. YZ, JL, and ZZ collected the clinical data. MQ and QZ supervised the project. All authors contributed to the article and approved the submitted version.

## Funding

This research was funded by 1.3.5 project for Disciplines of Excellence, West China Hospital, Sichuan University (ZYJC21042), Sichuan Science and Technology Programme (2019YFS0042) and the National Key Development Plan for Precision Medicine Research (2017YFC0910004).

## Conflict of Interest

The authors declare that the research was conducted in the absence of any commercial or financial relationships that could be construed as a potential conflict of interest.

## Publisher’s Note

All claims expressed in this article are solely those of the authors and do not necessarily represent those of their affiliated organizations, or those of the publisher, the editors and the reviewers. Any product that may be evaluated in this article, or claim that may be made by its manufacturer, is not guaranteed or endorsed by the publisher.
