# Supplementary material for: KRAS Gene Copy Number as a Negative Predictive Biomarker for the Treatment of Metastatic Rectal Cancer With Cetuximab: A Case Report
Source: Front Oncol. 2022 May 26;12:872630. doi: 10.3389/fonc.2022.872630 (PMC9207953; doi:10.3389/fonc.2022.872630)
Supplement: Supplementary file 1 [file Table_1.docx]

**Supplementary Table 1. Copy number of variation KRAS and mutant frequency of APC p.R499* during anti-tumor treatment and follow-up of the patient.**

| Date | Copy number variation of KRAS（n*） | APC p.R499* |
| --- | --- | --- |
| May 23, 2018 | 3.31 | 14.74% |
| August 23, 2018 | Not detected | 0.56% |
| November 6, 2018 | 6.58 | 42.06% |
| March 1,2019 | 2.9 | 9.99% |
| April 28, 2019 | 2.69 | 8.90% |
| September 4, 2019 | 3.48 | 15.10% |

* n: gene copy number, n>3 indicates gene copy number increased in tissue, n>2.5 indicates gene copy number increased in other samples.
